# A New Basal Ankylosaurid (Dinosauria: Ornithischia) from the Lower Cretaceous Jiufotang Formation of Liaoning Province, China

**DOI:** 10.1371/journal.pone.0104551

**Published:** 2014-08-13

**Authors:** Fenglu Han, Wenjie Zheng, Dongyu Hu, Xing Xu, Paul M. Barrett

**Affiliations:** 1 Faculty of Earth Sciences, China University of Geosciences (Wuhan), Wuhan, China; 2 Key Laboratory of Vertebrate Evolution and Human Origins of Chinese Academy of Sciences, Institute of Vertebrate Paleontology and Paleoanthropology, Chinese Academy of Sciences, Beijing, China; 3 University of Chinese Academy of Sciences, Beijing, China; 4 Paleontological Institute, Shenyang Normal University, Shenyang, China; 5 Department of Earth Sciences, Natural History Museum, London, United Kingdom; University of Pennsylvania, United States of America

## Abstract

A new ankylosaurid, *Chuanqilong chaoyangensis* gen. et sp. nov., is described here based on a nearly complete skeleton from the Lower Cretaceous Jiufotang Formation of Baishizui Village, Lingyuan City, Liaoning Province, China. *Chuanqilong chaoyangensis* can be diagnosed on the basis of two autapomorphies (glenoid fossa for quadrate at same level as the dentary tooth row; distally tapering ischium with constricted midshaft) and also a unique combination of character states (slender, wedge-like lacrimal; long retroarticular process; humerus with strongly expanded proximal end; ratio of humerus to femur length  = 0.88). Although a phylogenetic analysis places *Chuanqilong chaoyangensis* as the sister taxon of the sympatric *Liaoningosaurus* near the base of the Ankylosauridae, the two taxa can be distinguished on the basis of many features, such as tooth morphology and ischial shape, which are not ontogeny-related. *Chuanqilong chaoyangensis* represents the fourth ankylosaurid species reported from the Cretaceous of Liaoning, China, suggesting a relatively high diversity in Cretaceous Liaoning.

## Introduction

Ankylosauria is a group of quadrupedal herbivorous dinosaurs characterised by parasagittal rows of osteoderms on the dorsolateral surface of the body and a heavily armored skull [1]. The earliest records of the group have been reported from various Early or Middle Jurassic localities, and include *Bienosaurus lufengensis*, *Tianchisaurus nedegoapefererima*, and *Sarcolestes leedsi* [2]–[4], although all of these records have been considered either nomina dubia or dubiously referable to Ankylosauria [1], [5]. Definitive ankylosaur taxa are known to occur from the Late Jurassic (e.g., *Gargoyleosaurus* from western North America: [6]) to the end of the Cretaceous and their remains have been reported from all continents except Africa [1]. In Liaoning Province, China, three ankylosaurian species have been reported: *Liaoningosaurus paradoxus* from the Lower Cretaceous Yixian Formation [7] and *Crichtonsaurus bohlini* [8] and *C*. *benxiensis* [9] from the Upper Cretaceous Sunjiawan Formation. *Liaoningosaurus* was originally considered to be a possible nodosaurid [7], but a recent study suggests that it is a basal ankylosaurid [10]. *C*. *bohlini* and *C*. *benxiensis* are also referable to the Ankylosauridae (and are probably basal ankylosaurines) [9], [10].

Here, we describe a fourth ankylosaur species from Liaoning based on a specimen collected from the Lower Cretaceous Jiufotang Formation. This specimen preserves a nearly complete skeleton, and it provides new information on the morphology and taxonomy of the Ankylosauria.

## Materials and Methods

The permits for this research were obtained from the Chaoyang Jizantang Paleontological Museum of Liaoning, China. All of the materials described herein were collected from a single quarry by local farmers. Locality information was provided by staff at the Chaoyang Jizantang Paleontological Museum. The fossils are two-dimensionally preserved and visible in ventral view only. The material includes a skull and articulated postcranial material referable to a single individual. The skull and mandible are nearly complete, but have been strongly compressed dorsoventrally. The vertebral column includes the cervicals, dorsals, sacrals, and most of the caudals, but most of them are disarticulated. Both of the fore- and hind limbs are well preserved and articulated. Armor is preserved around the entire body but is only visible in ventral view.

In order to compare the ratios of humerus/tibia length to femur length with those of other ankylosaurs, the data were analysed in the software package SPSS 16.0 using the linear fit function. The best fit lines, regression equation and R^2^ values are presented in the Results.

The phylogenetic position of *Chuanqilong chaoyangensis* was inferred using parsimony analysis. The new taxon was incorporated into a previously published data matrix built to examine ankylosaurian interrelationships [10]. *Liaoningosaurus paradoxus* was also re-scored in the matrix based on our firsthand observations of the holotype specimen (Text S1). The modified data matrix consists of 170 characters and 52 taxa. The matrix was analyzed using TNT [11], and all of the characters were treated as equally weighted and unordered. The analysis was conducted using a heuristic search with 1000 replicates. TBR branch swapping was employed and 100 parsimonious trees were saved per replicate. A reduced consensus analysis was performed to identify wildcard taxa within TNT to provide maximum phylogenetic resolution for the new taxa [11]. Standard bootstrap values (absolute frequencies) were calculated using a traditional heuristic search with 1000 replications. Bremer supports were calculated by running the script “Bremer. run” automatically.

### Nomenclatural Acts

The electronic edition of this article conforms to the requirements of the amended International Code of Zoological Nomenclature (ICZN), and hence the new names contained herein are available under that Code from the electronic edition of this article. This published work and the nomenclatural acts it contains have been registered in ZooBank, the online registration system for the ICZN. The ZooBank LSIDs (Life Science Identifiers) can be resolved and the associated information viewed through any standard web browser by appending the LSID to the prefix “http://zoobank.org/”. The LSID for this publication is: urn:lsid:zoobank.org:pub:9D60475B-FA91-464B-8EBF-D335582AE23E. The electronic edition of this work was published in a journal with an ISSN (1932–6203), and has been archived and is available from the following digital repositories: PubMed Central (http://www.ncbi.nlm.nih.gov/pmc), LOCKSS (http://www.lockss.org).

### Institutional Abbreviations

**AMNH**, American Museum of Natural History, New York, USA; **BXGM**, Benxi Geological Museum, Liaoning Province, China; **CEUM**, Prehistoric Museum, College of Eastern Utah, Price, Utah, USA; **CJPM**, Chaoyang Jizantang Paleontological Museum; **DMNH**, Denver Museum of Natural History, Denver, Colorado, USA; **IVPP**, Institute of Vertebrate Paleontology and Paleoanthropology, Beijing, China; **LPM**, Liaoning Paleontological Museum, Shenyang, Liaoning Province, China; **MPC**, Mongolian Paleontological Center, Ulaanbaatar, Mongolia; **MTM**, Hungarian Natural History Museum, Budapest, Hungary; **MU**, University of Missouri, Columbia, Missouri, USA; **NHMUK**, Natural History Museum, London, UK; **PIN**, Paleontological Institute, Russian Academy of Sciences, Moscow, Russia; **QM**, Queensland Museum, Brisbane, Australia; **ROM**, Royal Ontario Museum, Toronto, Canada; **SMU**, Southern Methodist University, Dallas, Texas, USA; **TMP**, Royal Tyrrell Museum of Palaeontology, Drumheller, Alberta, Canada; **UALVP**, University of Alberta Laboratory for Vertebrate Paleontology, Edmonton, Alberta, Canada; **YPM**, Yale Peabody Museum, New Haven, Connecticut, USA.

## Results

### Systematic Paleontology

Dinosauria Owen, 1842 [12]

Ornithischia Seeley, 1887 [13]

Thyreophora Nopcsa, 1915 [14]

Ankylosauria Osborn, 1923 [15]

Ankylosauridae Brown, 1908 [16]

*Chuanqilong* **gen. nov. urn:lsid:zoobank.org:act:76DD6D3F-F23C-4AC0-B480-20AC73B50279**

#### Type Species

*Chuanqilong chaoyangensis* **gen. et sp. nov. urn:lsid:zoobank.org:act:EE6564A0-33AE-4CC7-B4EA-8CD682E1EB43**

#### Etymology

The generic name is derived from Chinese *Chuanqi* (legendary, referring to western Liaoning providing a spectacular assemblage of Mesozoic terrestrial fossils) + *long* (dragon). The specific name is derived from the broader geographical area including the type locality.

#### Holotype

CJPM V001, a nearly complete skeleton missing only the distal portion of the caudal series. The specimen is housed in the Chaoyang Jizantang Paleontological Museum. A cast of the holotype specimen is housed in the Institute of Vertebrate Paleontology and Paleoanthropology as IVPP FV 1978.

#### Locality and Horizon

**Figure 1:**
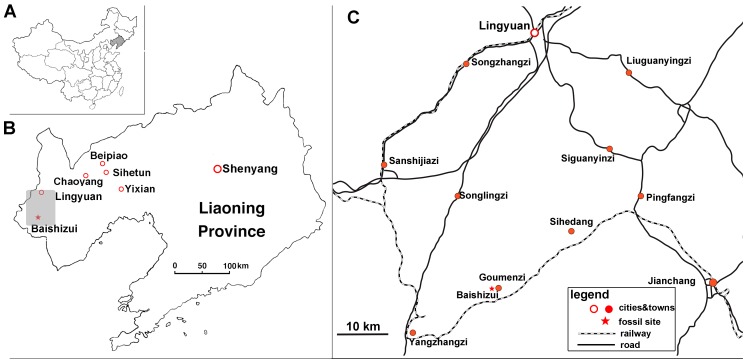
Locality maps. **A**, map of China showing location of Liaoning Province; **B**, enlarged map of Liaoning Province showing locality of fossil site, south of Lingyuan; **C**, enlarged map of Lingyuan city showing locality of fossil site at Baishizui village. [planned for page width].

**Figure 2:**
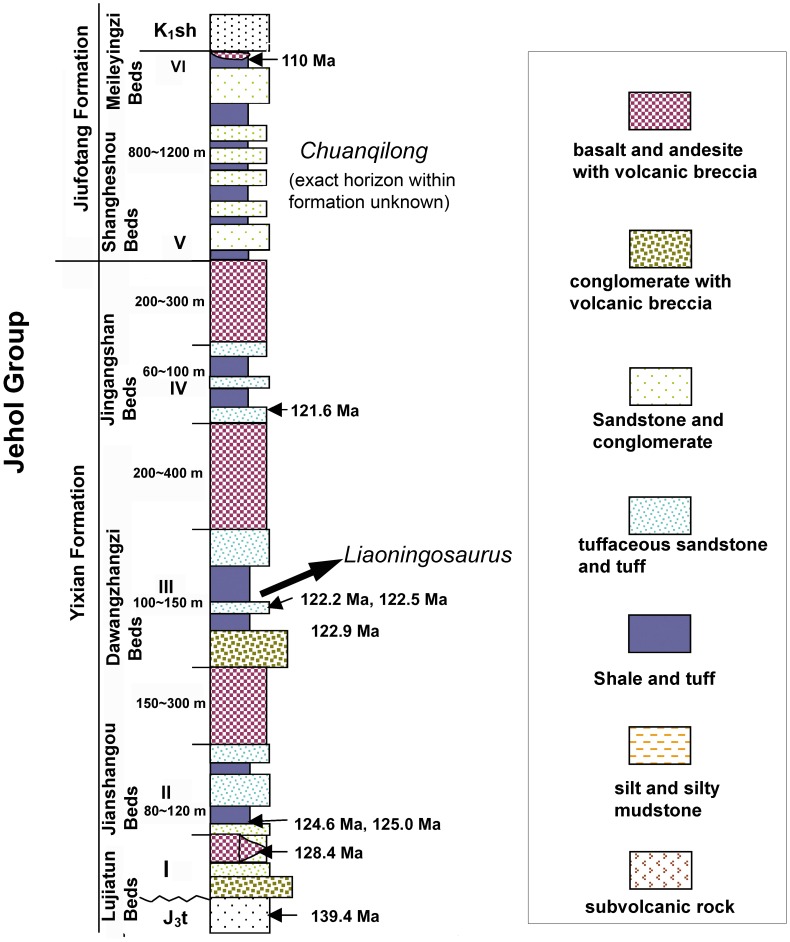
A stratigraphic column of the Jehol Group showing the position of *Liaoningosaurus* and *Chuanqilong*. Modified after [64]. [planned for page width].

Baishizui Village, Goumenzi County, Lingyuan City, Liaoning Province, China (Fig. 1); the quarry is in the Jiufotang Formation (Lower Cretaceous, Aptian) [17] (Fig. 2). A detailed stratigraphical investigation of this quarry is required to establish its relationships to other exposures of the Jiufotang Formation in the area.

#### Diagnosis

An ankylosaur distinguished from other ankylosaurs by two autapomorphies: the glenoid fossa for the quadrate is at the same level as the dentary tooth row; and the distally tapering ischium is constricted at midshaft length. *Chuanqilong* also differs from all other ankylosaurians in having the following unique combination of character states: presence of a long retroarticular process (differs from all other ankylosaurians except *Gargoyleosaurus*); presence of a slender, wedge-like lacrimal (differs from all other known ankylosaurians except *Minmi*); ratio of humerus to femur length of 0.88 (notably higher than in most known ankylosaurians except *Hungarosaurus* and *Liaoningosaurus*); the width of the proximal end of the humerus is half of the length of the humeral shaft (substantially different from that of *Liaoningosaurus*, in which this ratio is 0.38); presence of subtriangular unguals (absent in all other ankylosaurs except *Liaoningosaurus* and *Dyoplosaurus*).

### Description and Comparisons

**Figure 3:**
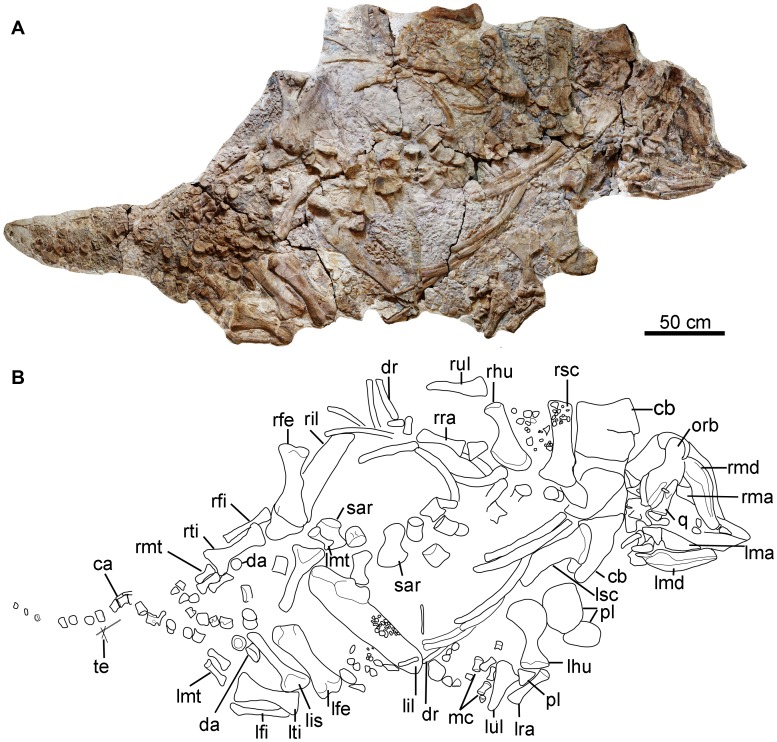
Photograph and outline drawing of the skeleton of *Chuanqilong chaoyangensis*. **A**, photograph; **B**, outline drawing. **Abbreviations**: **ca**, caudal vertebrae; **cb**, cervical band; **da**, dermal armor; **dr**, dorsal rib; **lfe**, left femur; **lfi**, left fibula; **lhu**, left humerus; **lil**, left ilium; **lis**, left ischium; **lma**, left maxilla; **lmd**, left mandible; **lmt**, left metatarsal; **lra**, left radius; **lsc**, left scapula; **lti**, left tibia; **lul**, left ulna; **mc**, metacarpals; **orb**, orbital; **pl**, plates; **q**, quadrate; **rfe**, right femur; **rfi**, right fibula; **rhu**, right humerus; **ril**, right ilium; **rma**, right maxilla; **rmd**, right mandible; **rmt**, right metatarsals; **rra**, right radius; **rsc**, right scapula; **rti**, right tibia; **rul**, right ulna; **sar**, sacral rib; **te**, tendons. [planned for page width].

The holotype skeleton is exposed mainly in ventral view (Fig. 3) and as result numerous anatomical details are not visible. In addition, the presence of some elements obscures large portions of the other bones present limiting the amount of information available. Nevertheless, preservation is generally good. Although the specimen represents a large animal (approximately 4.5 m in total body length; see Table 1 for measurements of the holotype skeleton), it is likely to be a juvenile individual based on several features. For example, the vertebral centra are not fused to their neural arches in all visible vertebrae including cervicals, dorsals, and caudals. In addition, the sacral ribs are not fused to the sacral centra. Consequently, an adult individual is likely to have been greater than 4.5 m in length. Jurassic ankylosaurians are mostly relatively small in size with body lengths no greater than 4 meters. For example, *Mymoorapelta* and *Gargoyleosaurus* each have lengths of approximately 3 m [18], [19]. By comparison, most Cretaceous ankylosaurians have body lengths greater than 5 m. For example, the primitive ankylosaurid *Cedarpelta* has an estimated body length of 7.5–8.5 m [20].

**Table 1:** Measurements of the girdles and limb bones of *Chuanqilong chaoyangensis*.

Bone	L/R	Length	Wp	Wd	Wm	1*	2*
Scapula	L	—	—	19.5	9	—	—
	R	40	—	19	9	—	—
Humerus	L	35	18	14	5.5	—	—
	R	35	—	—	—	20	—
Ulna	L	18	13	5	—	—	—
	R	17	13	6	—	—	—
Radius	L	23	6.5	6.5	4	—	—
	R	23	8	8	4	—	—
Femur	L	40	18	17	9	—	—
	R	40	20	18	9	—	—
Tibia	L	—	15	19	7	—	—
	R	36	12	17	6	—	—
Fibula	L	32	6	5	3.5	—	—
	R	35	6	5	3.5	—	—
Ilium	L	76	—	—	—	20	15
	R	—	—	—	—	20	15
Ischium	L	42	18	7	5	—	—
	R	41	19	7	5.5	—	—
Metacarpal I	L	7.5	4.5	4.5	—	—	—
Metacarpal II	L	8	4	4	—	—	—
Metacarpal III	L	8	4	5	—	—	—
Metacarpal IV	L	7	2	4	—	—	—
Metatarsal II	L	13	7.5	5	3.5	—	—
Metatarsal III	L	15.5	7.5	6	3.5	—	—
Metatarsal II	R	13	6.5	5	3.5	—	—
Metatarsal III	R	15	6	6	3	—	—
Metatarsal IV	R	13	5	6	1.5	—	—

*For humerus, 1) refers to the length from the proximal edge to the distal end of deltopectoral crest. For ilium, 1) refers to the length of the preacetabular process and 2 refers to the length of the postacetabular process.

### Skull

**Figure 4:**
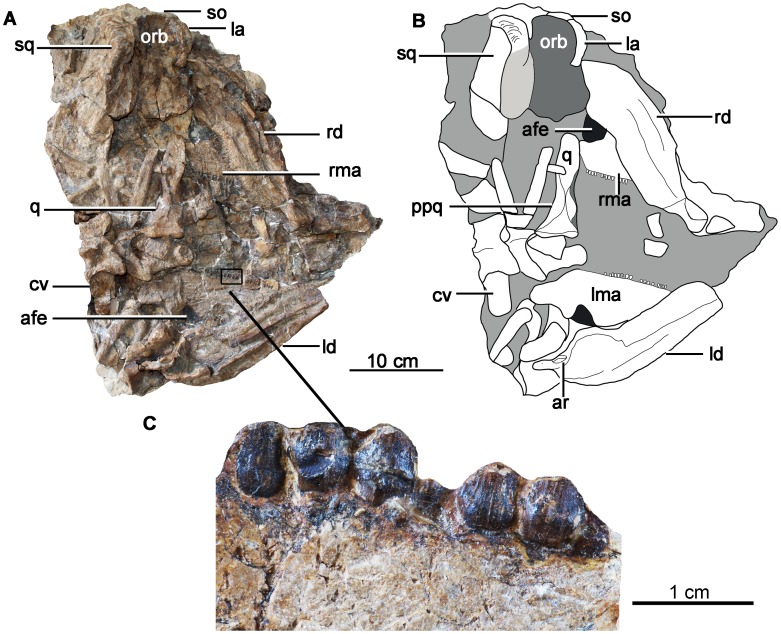
Holotype skull and mandibles of *Chuanqilong chaoyangensis*. **A**, photograph in ventral view; **B**, outline drawing in ventral view; **C**, close up to maxillary teeth in lateral view. **Abbreviations**: **afe**, antorbital fenestra; **ar**, articular; **cv**, cervical vertebra; **la**, lacrimal; **ld**, left dentary; **lma**, left maxillary; **orb**, orbital; **ppq**, pterygoid process of the quadrate; **q**, quadrate; **rd**, right dentary; **rma**, right maxilla; **so**, supraorbital; **sq**, squamosal. [planned for page width].

The skull and mandibles are strongly compressed dorsoventrally. The skull is triangular in ventral view, with a transverse width that was probably greater than its length, as in ankylosaurids (Figs. 3, 4) [21]. The maxilla is partially exposed in lateral view and exhibits a shallow, flattened buccal emargination. A large, triangular antorbital fossa or fenestra seems to be present, located in the caudodorsal region of the maxilla (Fig. 4A, B) in lateral view. An antorbital fenestra is also present in juvenile ankylosaurids such as *Liaoningosaurus* and *Pinacosaurus,* though it is replaced by a small concavity in adult *Pinacosaurus* [7], [22]. A small antorbital fenestra is also present in the probably adult *Minotaurasaurus* [22],[23], whereas it is either unknown or absent in all other ankylosaurians [1]. It seems likely that the presence of an open antorbital fenestra in *Chuanqilong chaoyangensis* is due to its juvenile status. However, the orbit is circular in outline, and relatively small in comparison to skull length, contrary to what would be expected in a juvenile individual. The lacrimal is slender and wedge-shaped (Fig. 4A, B), forming the rostral margin of the orbit, as in the basal ankylosaurid *Minmi*, whereas it is sub-rectangular in other known ankylosaurians, such as *Pinacosaurus* and *Cedarpelta* [1]. A long and dorsoventrally compressed supraorbital is visible in lateral view, contacting the lacrimal rostroventrally. Caudally, the postorbital is damaged and only partially exposed. The ‘squamosal’ may be composed of both the squamosal and a portion of the postorbital. It is subrectangular in outline and ornamented with subparallel grooves, as in *Minmi* [24] (Fig. 4A, B). The left quadrate is exposed in rostral view. It is long and straight with a rectangular head, and there is no indication that the quadrate head was fused with the squamosal, thereby differing from the condition seen in nodosaurids [25]. Below the quadrate head, the shaft is transversely expanded to form a wide, shallow, and rostrally-opening depression. The quadrate constricts ventral to this point and is narrowest at midshaft length. A crescentic depression is present on the cranioventral surface of the quadrate for reception of the quadratojugal. The pterygoid process is thin, short, and sub-triangular in outline. The transversely expanded ventral end is composed of two well-defined mandibular condyles, which are separated by a shallow groove ventrally. The medial condyle is transversely wider and extends further ventrally than the lateral condyle, as in most other ankylosaurians. No other features of the skull are visible.

### Mandible

The paired mandibular rami are preserved separately and both are visible in medial view only (Figs. 3, 4A, B). The predentary is missing. The mandible is long and shallow, as in other basal ankylosaurids, but it differs from other taxa in the apparent absence of an osteoderm from its ventral margin. The lack of an osteoderm in this area may be either due to incomplete preservation, suggesting that they were not fused to the mandibular bones and thereby supporting the suggestion that the holotype *Chuanqilong* was not fully grown. In adult individuals of other ankylosaurians, such as *Pinacosaurus* and *Saichania*, osteoderms are fused to the lateral surface of the mandible [22], [26]. Alternatively, an osteoderm, if present, might be restricted to the lateral-most corner of the mandible and hence obscured from view (this condition is present in juvenile individuals of *Pinacosaurus grangeri* e.g., IVPP V16853; V. Arbour, pers. comm.), which would also represent a juvenile feature. The dentary tooth row is straight or slightly arched dorsally, but it is not as strongly sinusoidal as those of derived ankylosaurians, such as *Euoplocephalus* [27] or *Pinacosaurus* [26]. In dorsal view, the rostral end of the dentary tooth row is curved medially. At least 20 alveoli are present. The ventral margin of the mandible is relatively straight in its rostral and middle regions, but curves caudodorsally in its caudal part. The right dentary symphysis is preserved and is slightly downturned, short, robust, and sub-triangular in cross-section. The Meckelian canal is open, long, and deep. The coronoid eminence is prominent and projects above the level of the dentary tooth row, as in nodosaurids, whereas the coronoid eminence is situated at approximately the same level as the dentary tooth row in ankylosaurids, including *Pinacosaurus* [26], *Euoplocephalus* [27], and *Ankylosaurus* [28]. The splenial and prearticular are missing, exposing the adductor fossa, which is large and located below the coronoid eminence. The articular is small and oval in outline in lateral view. The retroarticular process is long and slender, as in *Gargoyleosaurus*, but differs from those of all other ankylosaurians, which possess relatively short and deep retroarticular processes [19]. Unusually, the glenoid fossa is situated in a relatively dorsal position, lying at approximately the same level as the dentary tooth row, unlike the condition present in all other ankylosaurians known from appropriate material, in which the glenoid fossa is situated at a level ventral to the dentary tooth row (e.g., *Pinacosaurus*: [26]). Unfortunately, this feature cannot be assessed in *Liaoningosaurus* [7].

### Dentition

Premaxillary teeth are unknown due to breakage of the snout. Both maxillary and dentary teeth are preserved, with the former exposed labially and the latter exposed lingually.

There are at least 20 alveoli in the left maxilla. The maxillary tooth counts of most ankylosaurians are around 20: *Ankylosaurus* has the largest number of maxillary teeth (34–35) [28], whereas *Liaoningosaurus* has the smallest number (about 10: [7]). Tooth count probably increases during growth [7] and adult tooth count also varies between species [28]. The teeth and their marginal denticles are small in comparison to the size of skull, as occurs typically in ankylosaurids [21].

The four preserved rostral maxillary teeth are smaller than the caudal teeth. The crowns are as tall as their width with sub-triangular outlines, as in most ankylosaurians (Fig. 4C). The base of the crown is strongly swollen with a weak cingulum, as in ankylosaurids [21]. There is no shallow notch at the base of tooth crowns, unlike the notched condition present in the ankylosaurid *Crichtonsaurus* [8] and the nodosaurid *Edmontonia* [29]. Additionally, the teeth of *C. bohlini* bear much larger denticles and a more distinct cingulum than those of *Chuanqilong*. There is no distinct primary ridge, and secondary vertical ridges and grooves are present on the labial surfaces of the tooth crowns. These ridges usually terminate apical to the cingulum, but some ridges extend across the cingulum to the basal margin of the crown. The crescentic cingula seen in some ankylosaurs, such as *Texasetes*, are absent. Small denticles and cusps are present on one rostral maxillary tooth crown. The denticles are small and tapering with a round cross-section at their base. However, most of the teeth do not bear these structures, though it is not clear if this absence is due to poor preservation or tooth wear. Most of the dentary teeth are missing. Those that are preserved are only partially exposed in the left dentary. Dentary teeth seem to have been similar in size and shape to the maxillary teeth.

### Axial Skeleton

Several cervical and dorsal vertebrae are scattered on the slab. The cervical centra are spool-like and shorter than they are wide: their articular surfaces are all obscured. The dorsal centra are also spool-like in ventral view, slightly amphicoelous, and possess concave lateral surfaces. Dorsal centra are longer than tall. A ventral keel is absent from all of the preserved dorsal centra. One sacral vertebra is exposed on the slab in ventral view. Its centrum is wider than it is long, and its exposed articular surface is rugose, suggesting that it had not yet fused with the other sacrals. Several sacral ribs are preserved separately. They are robust and dumbbell-shaped in outline. Approximately 20 caudal vertebrae are preserved, but most of them are disarticulated. The centra of the proximal and middle caudal vertebrae are shorter than they are wide. Deep longitudinal grooves are present on the ventral surfaces of the proximal caudal vertebrae. Chevron facets are well developed, with the caudal facets more prominent than the cranial ones.

One middle caudal vertebra is well preserved. Its centrum is relatively longer and transversely narrower than those of the cranial caudals and in lateral view it has a square outline. The transverse process is reduced to a small nodular process and is located on the dorsal part of the lateral surface of the centrum. The neural spines are elongate, oriented caudodorsally, and possess arc-shaped outlines. The prezygapophyseal facets are oval in outline and face craniomedially, whereas the postzygapophyses are positioned near the tip of the neural spine and face caudolaterally.

Caudually, three additional distal caudal vertebrae are tightly articulated. Their centra are more elongate and transversely narrower than those of the proximal and middle caudals. In these vertebrae, the neural spine has merged with the postzygapophyses to form a single midline caudal process that extends caudally. The caudal process terminates cranial to the midpoint of following vertebra, as in nodosaurids, but unlike the condition in derived ankylosaurids, in which the process is longer [21]. The prezygapophyses are short and reduced in size, corresponding to the size reduction of the postzygapophyses. No chevrons are preserved.

Several ossified hypaxial tendons are present near the distal region of the tail (Fig. 3). During preservation, they have moved from their original positions and arrangement so that they are now aligned in different orientations. On the basis of the morphology of the preserved caudals, which does not conform with that of the tail club handle morphology seen in ankylosaurine taxa, a tail club knob was probably absent, as in all nodosaurids and some basal ankylosaurids (e.g., *Minmi*: [30]). Presence of a tail club was formerly an important diagnostic feature of ankylosaurids [21], but recent work has indicated that tail clubs may have been present in ankylosaurines only, a clade that includes *Ankylosaurus* [28], *Euoplocephalus* [31], *Pinacosaurus* [32], *Talarurus* [33], *Saichania* [34], *Tarchia* [35], *Tianzhenosaurus* [36], *Dyoplosaurus* [37], and *Nodocephalosaurus* [38]. It is unknown whether tail clubs were present or not in the basal ankylosaurids *Crichtonsaurus* [8], [9], *Cedarpelta* [20], *Gobisaurus* [39], and *Shamosaurus*, but the ankylosaurids *Minmi*, *Liaoningosaurus*, and *Zhongyuansaurus* lack tail clubs [7], [40], [41]. The likely absence of a tail club in *Chuanqilong chaoyangensis* adds further support to the hypotheses that the tail club is a derived feature that appears only in derived, and currently only Late Cretaceous taxa.

### Pectoral Girdle and Forelimb

#### Scapula

**Figure 5:**
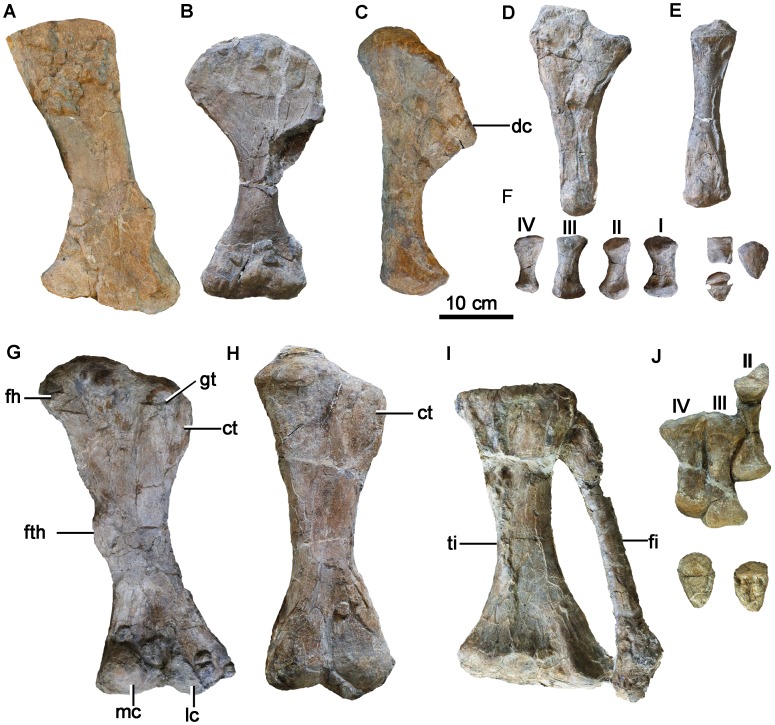
Postcranial materials of *Chuanqilong chaoyangensis*. **A**, right scapula in lateral view; **B**, left humerus in cranial view; **C**, right humerus in lateral view; **D**, left ulna in medial view; **E**, left radius in medial view; **F**, disarticulated left metacarpals and phalanges; **G**, right femur in caudal view; **H**, left femur in cranial view; **I**, articulated left tibia and fibula in cranial view; **J**, right metatarsals in cranial view; unguals in both cranial and caudal view. Note that due to compression of the right femur, the cranial trochanter is visible in posterior view whereas it would normally be obscured. **Abbreviations**: **dc**, deltopectoral crest; **fh**, femoral head; **fi**, fibula; **fth**, fourth trochanter; **gt**, greater trochanter; **lc**, lateral condyle; **ct**, cranial trochanter; **mc**, medial condyle; **ti**, tibia. [planned for page width].

Both scapulae are preserved. The scapula and coracoid are not co-ossified, contrary to the condition in most ankylosaurians (e.g., *Ankylosaurus*: [28]), but this may be another indication of specimen immaturity [42]. The scapula and coracoid are unfused in all known juvenile ankylosaurs, including *Liaoningosaurus* [7], an indeterminate nodosaurid hatchling from the Paw Paw Formation of Texas, juvenile *Pinacosaurus* [43], and *Anoplosaurus* [42], but they are fused in most adult or sub-adult individuals (Table 2). The scapula blade is slender, deflected caudoventrally, and has a rhomboidal outline (Figs. 3, 5). The dorsal margin of the scapula blade is relatively straight, whereas its ventral margin is concave. The caudal margin expands dorsoventrally. The shaft of the scapula blade is narrowest caudal to the glenoid fossa. A transverse flange is positioned along the craniodorsal margin of the scapula, as in ankylosaurids, whereas in nodosaurids the acromion is positioned ventrally, near the glenoid, and overhangs the coracoid [21]. There is no distinct enthesis present on the ventral edge of the scapula, which probably marks the insertion of the M. triceps longus caudalis (See [28]), and has been reported in derived ankylosaurids, including *Crichtonsaurus* [9], *Ankylosaurus* [28], and *Euoplocephalus* [31]. The absence of a distinct enthesis also suggests that CJPM 001 is not fully grown (K. Carpenter, pers. comm.). The glenoid fossa is large, oval in outline, and faces ventromedially. Both of the coracoids are missing or concealed.

**Table 2:** Measurements and comparisons in various ankylosaurians, in centimeter.

Taxon	Specimen number	Femur length	Tibia length	Humerus length	Scapula & coracoid	Ratio H/F	Ratio T/F	Reference
*Liaoningosaurus paradoxus*	IVPP V12560	2.5	2.5	2.5	unfused	1	1.00	[7]
nodosaurid scuteling from Paw Paw Formation	SMU 72444	7.3	–	6.84	unfused	0.94	–	[52]
*Anodontosaurus lambei*	AMNH 5266	25.5	190	–	unknown	–	0.75	[31]
*Crichtonsaurus benxiensis*	BXGMV0012-1	32	29	23	fused	0.72	0.91	[9]
*Crichtonsaurus bohlini*	LPM 101	34.3	–	24	unfused	0.70	0.00	[8]
*cf. Pinacosaurus*	MPC 100/1305	38	20.3	26.5	–	0.70	0.53	[66]
***Chuanqilong chaoyangensis***	CJPM 001	40	36	35	unfused	0.88	0.90	this study
*Pinacosaurus granger*	PIN 614	40	27	30	unknown	0.75	0.68	[66]
*Animantarx ramaljonesi*	CEUM 6288R	41.5	–	29.8	fused	0.72	–	[20]
*Gargoyleosaurus parkpinoorum*	DMNH 27726	46.5	–	29.2	unknown	0.63	–	[19]
*Talarurus plicatospineus*	PIN 557-3	47	24.8	33.5	fused	0.71	0.53	[33]
*Hungarosaurus tormai*	MTM 2007.25	49	–	45.5	fused	0.93	–	[55]
*Euoplocephalus tucki*	UALVP 31	51.5	–	37.7	probablyunfused	0.73	–	[31]
*Euoplocephalus tucki*	AMNH 5404	53.5	42.1	40.3	fused	0.75	0.79	[31]
*Polacanthus foxii*	NHMUK R175	53	34.5	–	unknown	–	0.65	[53]
*Dyoplosaurus acutosquameus*	ROM 784	56.2	–	–	unknown	–	–	[37]
*Scolosaurus cutleri*	NHMUK nR5161	60	41.5	44	fused	0.73	0.69	[14]
*Ankylosaurus magniventris*	AMNH 5214	67	–	54.2	fused	0.81	–	[28]
*Sauropelta edwardsi*	AMNH 3036	70	–	49.5	fused	0.71	–	[67]

#### Humerus

**Figure 6:**
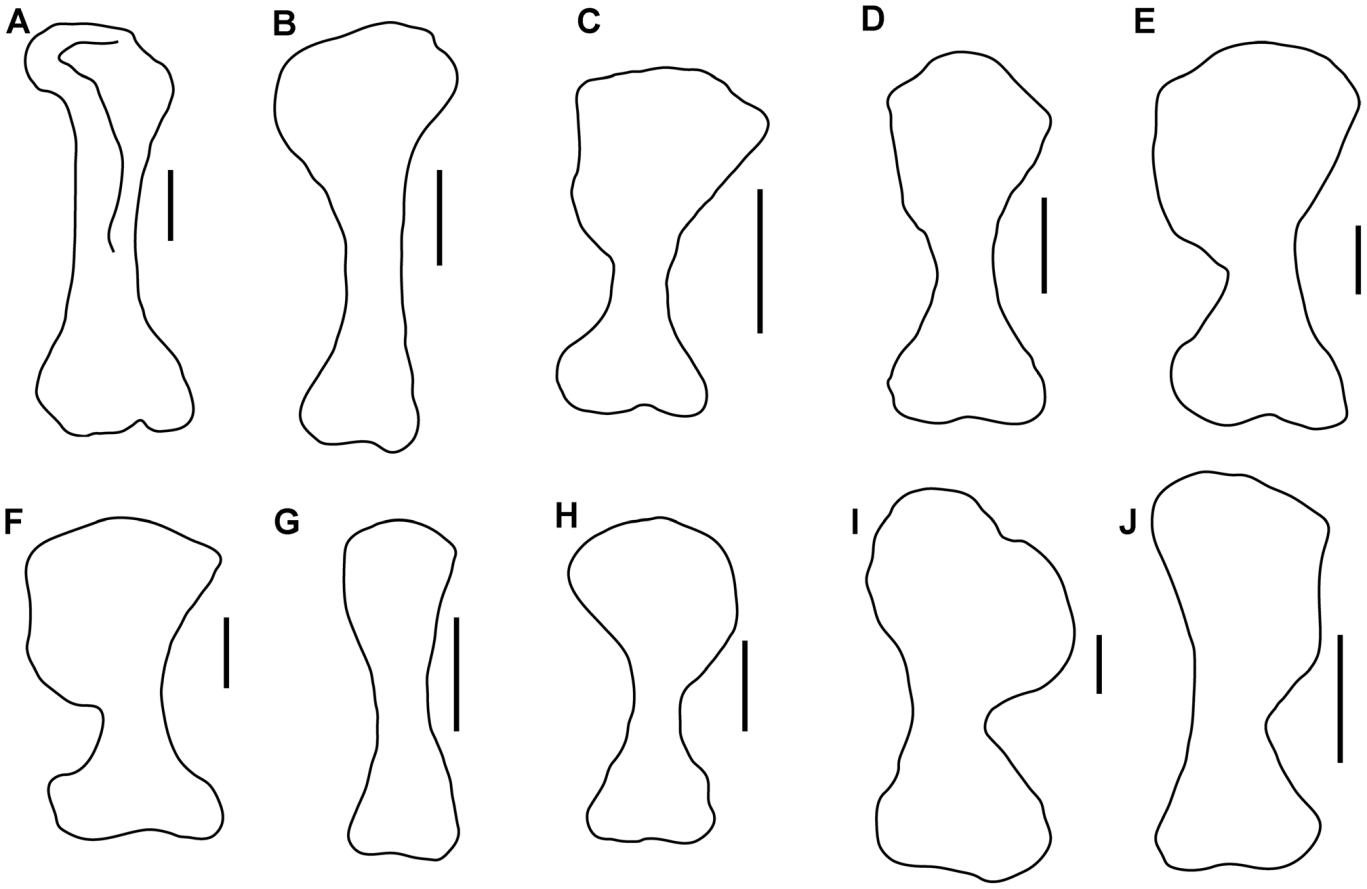
Comparison of outline drawings of ankylosaur humeri. A–G, right humeri in cranial view. A, *Sauropelta edwardsi* (YPM 5179), redrawn from [21]; B, *Hungarosaurus tormai* (MTM 2007.25.3), redrawn from [55]; C, *Crichtonsaurus benxiensis* (BXGMV0012), redrawn from [9]; D, *Niobrarasaurus coleii* (MU 650 VP), redrawn from [65]; E, *Ankylosaurus magniventris* (AMNH 5214), redrawn from [28]; F, *Euoplocephalus tutus* (AMNH 5337), redrawn from [31]; G, *Liaoningosaurus paradoxus* (IVPP V12560); H–I, left humerus in cranial view. H, *Chuanqilong chaoyangensis*; I, *Cedarpelta bilbeyhallorum* (CEUM 11629), redrawn from [44]; J, *Pinacosaurus* (MPC 100/1310), redrawn from [46]. Scale bars in A–F, H–I equal 10 cm; Scale bar in G equals 1 cm; scale bar in J equals 5 cm. [planned for page width].

Both humeri are well preserved, except that the ventral part of the deltopectoral crest is damaged on the left humerus. The left humerus is exposed in cranial view, and the right humerus in lateral view (Figs. 3, 5B, C). The humerus is short and robust. The deltopectoral crest is large and rounded in outline in cranial view, unlike in *Crichtonsaurus benxiensis* which possesses a straight lateral margin [9] (Fig. 6C). The deltopectoral crest and the transverse axis through the distal condyles are in the same plane, and the deltopectoral crest extends for more than half of the length of the humerus as in ankylosaurids, but unlike the condition in nodosaurids, which possess a relatively short deltopectoral crest [21]. There is no distinct separation between the humeral head and the deltopectoral crest as in most ankylosaurians, but in contrast to *Cedarpelta* and *Ankylosaurus* in which the dorsal surface of the deltopectoral crest is lower than the humeral head [28], [44] (Fig. 6). The width of the proximal end is much greater than the distal width as in most ankylosaurids except *Zhongyuansaurus*, in which both ends are of equal width [40]. The laterally placed radial condyle is oval and more prominent than the medial ulna condyle. The lateral epicondylar ridge is not well developed.

#### Ulna and Radius

**Figure 7:**
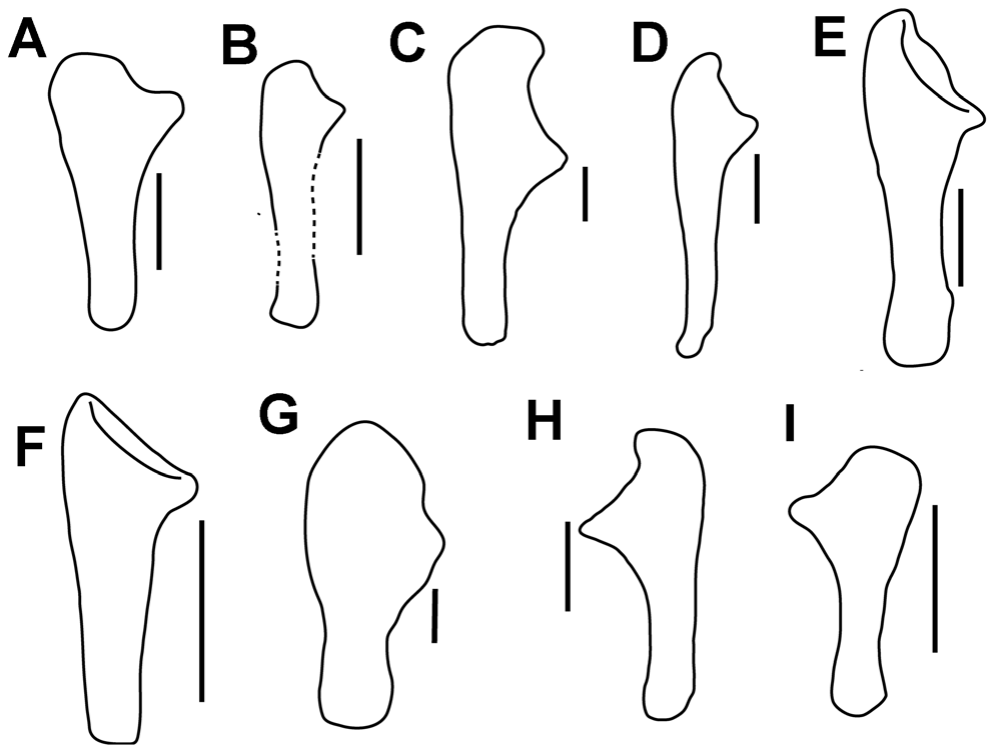
Comparison of outline drawings of ankylosaur ulnae. A, *Chuanqilong chaoyangensis*, left ulna in medial view; B, *Liaoningosaurus paradoxus* (IVPP V12560), left ulna in medial view; C, *Peloroplites cedrimontanus* (CEUM 11347), right ulna in lateral view, from [44]; D, *Hungarosaurus tormai* (MTM 2007.25.2), right ulna in lateral view, from [55]; E, *Niobrosaurus coleii* (MU 650 VP), right ulna in lateral view, from [65]; F, *Minmi* sp. (QMF 18101), left ulna in medial view, from [24]; G, *Cedarpelta bilbeyhallorum* (CEUM 10256), left ulna in medial view, from [44]; H, *Euoplocephalus tutus* (AMNH 5406), right ulna in medial view, from [31]; I, *Pinacosaurus* (MPC 100/1323), left ulna in lateral view, from [46]. Scale bars in A, C–H equal10 cm; scale bar in B equals 1 cm; scale bar in I equals 5 cm. [planned for page width].

Both of the ulnae and radii are complete (Figs. 3, 5D, E). The olecranon process of the ulna is low and wedge-shaped as in *Liaoningosaurus* [7] and juvenile specimens of *Pinacosaurus* [45], whereas it is tall and strongly developed in most large ankylosaurians, such as *Pelorolites* and *Cedapelta* [44] (Fig. 7). The low olecranon process may represent an ontogenetically variable character of ankylosaurians [7]. The humeral notch is moderately developed. The radius is slender in comparison with the ulna. It is rod-like with a flat proximal articular surface and a rugose, convex distal end. The distal end is wider transversely than the proximal end.

#### Manus

The left manus contains four complete but disarticulated metacarpals and their identifications are based on the well preserved metacarpals of *Peloroplites* [44] and *Pinacosaurus* [45], [46] (Fig. 5F). All of the preserved metacarpals are slender, as in *Pinacosaurus* [45]. Metacarpal III is the longest. Metacarpals I and II are sub-equal in length. Metacarpal IV is significantly shorter than other metacarpals. Metacarpal I is the most robust of the metacarpals, as in other ankylosaurids [1]. Metacarpals II and IV are more slender than metacarpals I and III. All of the metacarpals have expanded proximal and distal ends. The proximal articular surfaces are slightly concave, whereas the distal articular surfaces are strongly convex. There are no distinct ginglymi at the distal end. The phalanges are proximodistally short and transversely wide. The ungual phalanges are triangular in outline with sharp point in dorsal view. Their ventral surfaces are flattened and their proximal surfaces have a round outline and are slightly concave.

### Pelvic girdle and hind limb

#### Ilium

**Figure 8:**
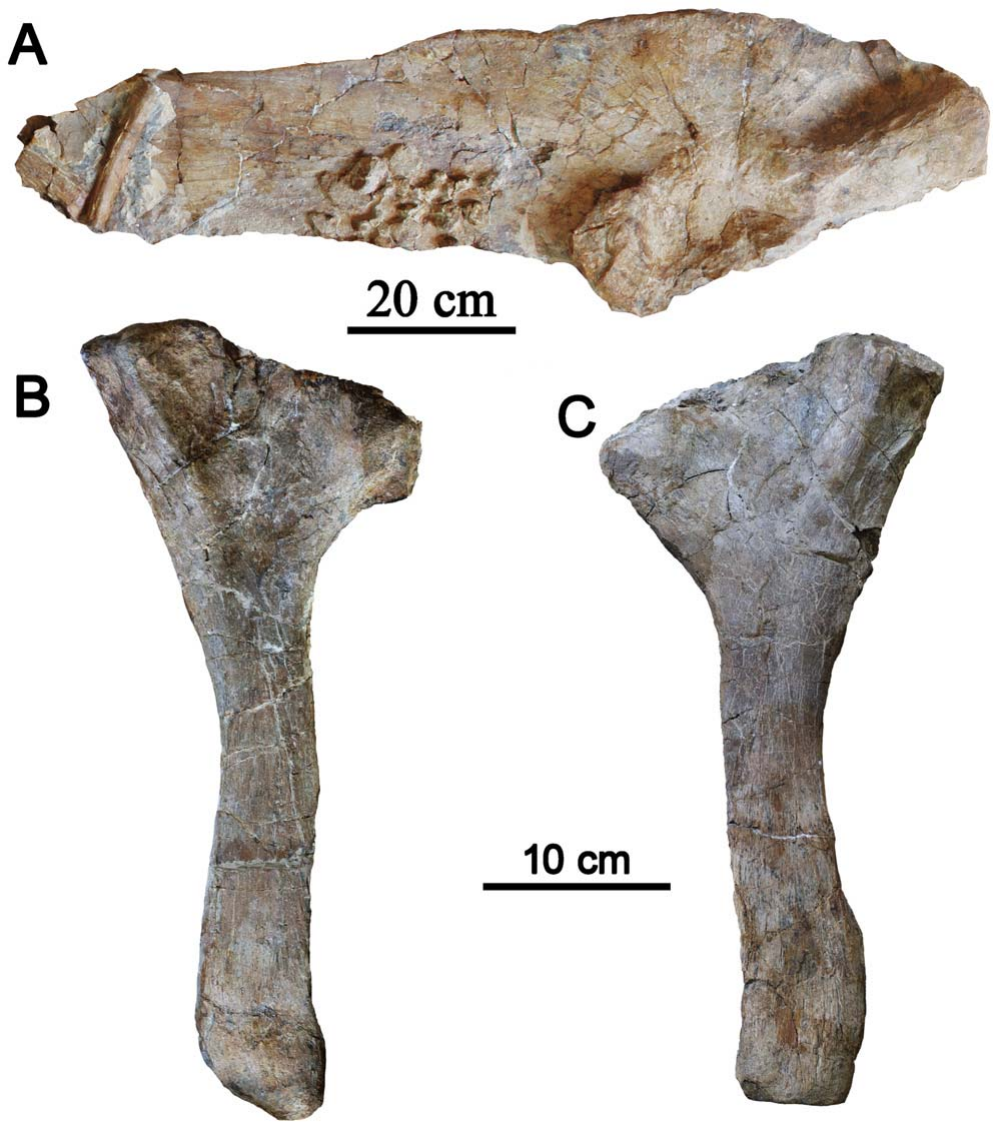
Left ilium and Ischia of *Chuanqilong chaoyangensis* in lateral view. **A**, left ilium in ventral view; B, right ischium in lateral view; **C**, left ischium in lateral view. [planned for column width].

Both ilia are well preserved and exposed in ventral view (Figs. 3, 8). As in other ankylosaurs, the preacetabular process rotated medially, making the ‘original’ lateral surface face dorsally, whereas the postacetabular process rotated in apposition and the original surface faces ventrally [47]. The preacetabular process is very long and transversely wide, and diverges laterally from the vertebral column at an angle of approximately 45°. The lateral margin of the preacetabular process is straight in ventral view, as in ankylosaurids, but unlike the condition in most nodosaurids, such as *Sauropelta* [25], *Struthiosaurus* [48], and *Zhejiangosaurus* [49], in which it is laterally curved. The postacetabular process is subtriangular in outline. It is very short, with a length less than that of the acetabulum, as in ankylosaurids [1]. The acetabulum is imperforate with a concave articular surface for accepting the femoral head, as in all ankylosaurians except *Mymoorapelta* [18]. The pubic peduncle is well developed with a sub-rounded profile and is dorsoventrally compressed. The ischial peduncle is rudimentary.

#### Ischium

**Figure 9:**
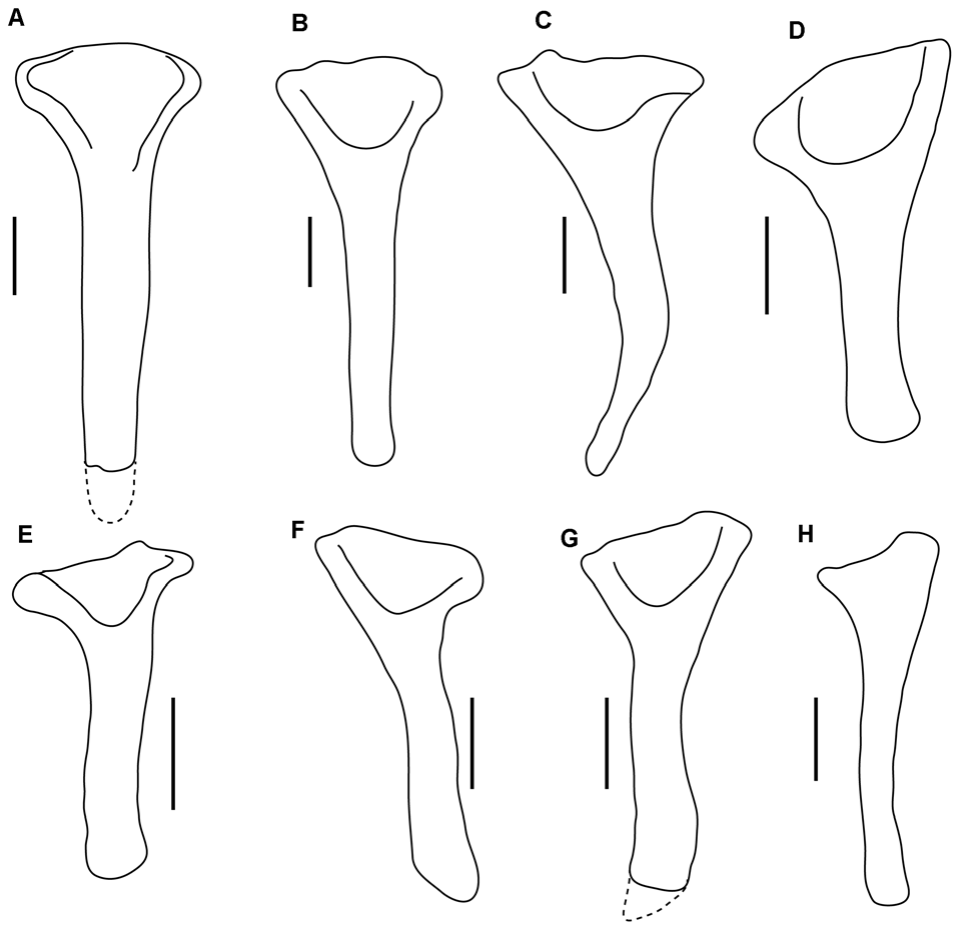
Comparison of outline drawings of ankylosaur ischia. A–F, left ischium in lateral view. A, *Ankylosaurus magniventris* (AMNH 5214), redrawn from [28]; B, *Scolosaurus cutleri* (TMP 2001.42.19), redrawn from [31]; C, *Edmontonia rugosidens*, redrawn from [25]; D, *Cedarpelta bilbeyhallorum* (CEUM 10266) from [44]; E, cf. *Pinacosaurus*, MPC 100/1305 in lateral view, from [22], [66]; F, *Chuanqilong chaoyangensis* in lateral view; G–H, right ischium in lateral view; G, *Chuanqilong chaoyangensis*; H, *Liaoningosaurus paradoxus* (IVPP V12560). Scar bars in A–G equal 10 cm; scale bar in H equals 1 cm. [planned for column width].

The ischium is long, slender, and mediolaterally compressed (Fig. 8B, C). The proximal end is expanded craniocaudally and contributes to half of the medial wall of the shallow acetabulum. There is no obturator process, which is absent in all ankylosaurians. The shaft of the ischium is slender and slightly curved ventrally, as in the ankylosaurid *Zhongyuansaurus* [40], whereas it straight in most ankylosaurids [28] and significantly curved ventrally near the distal end in nodosaurids [1]. The shaft of the ischium is unique in being narrower in its mid-shaft region and widening towards to the distal end, prior to tapering again further distally, whereas in other ankylosaurians the shaft either tapers distally along the whole shaft (e.g. *Ankylosaurus* [28], *Sauropelta*, *Edmontonia* [48]) or remains sub-equal in size along the whole shaft (e. g., *Euoplocephalus*: [31]), or is just slightly expanded at the distal end (e.g., *Cedarpelta*: [44]) (Fig. 9). The proximal end of the ischium is straight in lateral view. This is unlike the convex and fan-like ischium in the ankylosaurids *Ankylosaurus* [50] and *Euoplocephalus* [51], and also unlike the concave proximal ischia of the nodosaurids *Struthiosaurus* and *Edmontonia* [48]. The proximal end of the ischium lacks the medial wall present in the basal ankylosaurid *Cedarpelta* [20], [44].

#### Femur

The femur is robust and straight, as in other ankylosaurians (Figs. 3, 5G, H). The femoral head is robust and expanded forming a spherical articular surface. It forms an angle of about 145° with the long axis of the femur. Both the cranial and greater trochanters are present, and they are separated from the femoral head by a prominent constriction. The cranial trochanter is slender, finger-like, and separated from the greater trochanter by a vertical cleft. The cranial trochanter is present in juveniles, such as the Paw Paw nodosaurid scuteling and *Anoplosaurus*, but fused with the greater trochanter in most adult ankylosaurs [52]. However, the cranial trochanter is also present in some large primitive nodosaurids, such as *Polacanthus* [53] and *Texasetes* [54]. So the presence of a cranial trochanter is likely to be a primitive character of ankylosaurids, as well as being under ontogenetic control in some taxa. The fourth trochanter is a prominent rugosity that is located distal to femoral mid-length, as in typical ankylosaurids [21].

**Figure 10:**
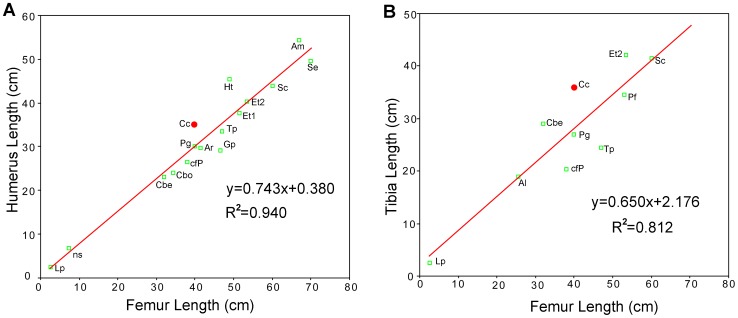
Differential forelimb and hind limb measurements across ankylosaurs. Symbols for *Chuanqilong* are in red. A, plot of humeral length versus femoral length. Values for *Chuanqilong* is fall well below the line of best fit; B, plot of tibial length versus femoral length. **Abbreviations**: **Al**, *Anodontosaurus lambei*; **Am**, *Ankylosaurus magniventris*; **Ar**, *Animantarx ramaljonesi*; **Cbe**, *Crichtonsaurus benxiensis*; **Cbo**, *Crichtonsaurus bohlini*; **Cc**, *Chuanqilong chaoyangensis*; **cfP**, *cf. Pinacosaurus*; **Et1**, *Euoplocephalus tucki* UALVP 31; **Et2**, *Euoplocephalus tucki* AMNH 5404; **Gp**, *Gargoyleosaurus parkpinoorum*; **Ht**, *Hungarosaurus tormai*; **Lp**, *Liaoningosaurus paradoxus*; **ns**, nodosaurid scuteling from Paw Paw Formation; **Pf**, *Polacanthus foxii*; **Pg**, *Pinacosaurus grangeri*; **Sc**, *Scolosaurus cutleri*; **Se**, *Sauropelta edwardsi*; **Tp**, *Talarurus plicatospineus*. [planned for page width].

Distally, a shallow cranial intercondylar fossa is present. A deep caudal intercondylar groove divides the medial (tibia) and lateral (fibula) condyles, and the former is slightly larger than the latter. The ratio of humerus to femur length is 0.83, similar to the condition in *Ankylosaurus*, but lower than the ratios in *Liaoningosaurus*, the indeterminate juvenile nodosaurid from Paw Paw Formation, and *Hungarosaurus*, and greater than those of other known ankylosaurians (Table 2; Fig. 10). Juveniles may have proportionally longer forelimbs than adults [52]. The juvenile *Liaoningosaurus* and indeterminate Paw Paw nodosaurid have humerus to femur length ratios of 1.0 and 0.93, respectively, whereas the ratio is about 0.7 in most large ankylosaurians (Table 2). However, this ratio is also substantially higher in adult *Hungarosaurus* (0.92: [55]), *Ankylosaurus* (AMNH 5214, 0.81, [28]), and in the late juvenile *Chuanqilong* (0.88) (Fig. 10A). This suggests that the ratio of humerus to femur length may represent a valid taxonomic difference.

#### Tibia

The tibia is shorter than the femur (Figs. 3, 5I). The ratio of tibia to femur length is approximately 0.9. This is similar to the ratio in *Crichtonsaurus benxiensis* (0.91: [9]), *Europelta carbonensis* (0.91: [56]), *Liaoningosaurus paradoxus* (0.95: pers. observ.) and greater than in all other known ankylosaurians (Table 2; Fig. 10B). The tibia is straight, robust, and greatly expanded mediolaterally both proximally and distally. The transverse expansion of the proximal end is relatively weaker than that of the distal end in cranial view.

#### Fibula

The fibula is slender and slightly shorter than the tibia. The proximal end is expanded craniocaudally and compressed laterally. The whole shaft is relatively equal in size and oval in cross-section. The distal end is slightly expanded mediolaterally with a flattened cranial surface.

#### Proximal tarsals

The calcaneum and astragalus are not preserved, and they are inferred to have remained unfused to the distal end of the tibia. The calcaneum and astragalus are usually fused with the distal end of the tibia in most ankylosaurians [1], but they are unfused in juveniles of *Anodontosaurus lambei* (AMNH 5266; [31]) *Liaoningosaurus* [7], and *Pinacosaurus* [46], suggesting that this was under ontogenetic control in ankylosaurians. However, the astragalus is not fused to the distal end of the tibia in the early ankylosaurians *Mymoorapelta* (DMNH 15162: [57]), *Peloroplites* (CEUM 11319: [44]), and possibly *Hylaeosaurus* (NHMUK R2615: [53]), which indicates they may have been unfused primitively in adult ankylosaurians.

#### Pes

Metatarsals II, III, and IV are well preserved and in articulation in the right foot (Figs. 3, 5J). The possible presence of metatarsals I and V cannot not be excluded due to the preservation of the specimen. Metatarsal III is much longer (187.5% of the length) and more robust than metacarpal III. This ratio is similar to that seen in the primitive ankylosaurid *Gargoyleosaurus* (184.4%: [19]), greater than in most ankylosaurians, such as *Pinacosaurus grangeri* (113.5%: [45]) and *Talarurus plicatospineus* (132.8%: [45]), but smaller than that in *Liaoningosaurus*, which has even longer metatarsals (more than 200%: [7]). Metatarsals II and IV are sub-equal in length, and metatarsal III is longer and more robust than the other two metatarsals. They all have expanded proximal and distal ends. The proximal end of metatarsal II is dorsoventrally deeper than it is wide transversely and it has a concave and oval articular surface for the distal tarsals. Metatarsal III has a square cross-section proximally, and metatarsal IV is transversely wider than deep craniocaudally. The distal ends of all metatarsals are transversely expanded and bear no, or very weak, ginglymi.

The unguals are robust and sub-triangular in outline with sub-rounded distal ends in dorsal view. This is unlike the pedal unguals of *Liaoningosaurus*, which have much sharper distal ends [7]. Coombs [51] noted that in ankylosaurs the pedal unguals are widest at a point approximately one-third of the distance from their proximal ends in juveniles, whereas in adults they are widest proximally. However, in juveniles like *Liaoningosaurus* and *Chuanqilong*, the pedal unguals are widest proximally and taper distally, contrary to this observation. Ungual shape may, therefore, be a useful character for taxonomic or systematic purposes, and similar sub-triangular unguals are also known in *Dyoplosaurus* [37].

### Armor

Cranial armor is not visible on the slab. The cervical armor usually comprises two cervical half rings in ankylosaurids and three cervical half rings in nodosaurids. These half rings consist of a superficial layer of osteoderms fused to an underlying band of bone. The osteoderms are usually pitted and rugose, similar to body osteoderms, whereas the connecting band is usually smooth and plate-like [58]. In *Chuanqilong*, only one cervical half ring is present in ventral view. The band appears to be fused into a single large plate, as in ankylosaurids [58]. However, it is compressed dorsoventrally, damaged and separated into four sections (Fig. 3). The right three sections are thickened, arched dorsally, and subrectangular in outline with smooth ventral surfaces. The left section has a wide medial edge and tapers caudolaterally with a subtriangular profile, and this section has a straight craniolateral edge as also seen in *Gargoyleosaurus* [19], but which is unknown in other ankylosaurians. Two large armor plates are preserved in the shoulder region. They are thickened, subrectangular in outline, have flat, smooth surfaces, and are thicker along their margins than centrally. The larger plate is twice the length of the smaller one. Both of them are similar to the osteoderms of the cervical half ring, and they may represent separate cervical armor plates. A relatively small triangular armor plate is present between the proximal end of the left ulna and radius (Fig. 3). It is dorsoventrally compressed, wide at the base and tapers distally. One nearly complete oval armor plate is present near the left ischium in dorsal view, which is sharply keeled along its midline (Fig. 3). A variety of small irregular osteoderms and ossicles are preserved over the whole body in ventral view (Fig. 3), as in most ankylosaurians. Dermal armor is absent in the indeterminate Paw Paw nodosaurid ([52]: SMU 72444), the juvenile specimen of *Anoplosaurus* [42], and the hatchling dinosaur *Propanolosaurus* [52], [59]. However, dermal armor is present in the small specimen *Liaoningosaurus*, suggesting that the ability to produce dermal armor had already appeared by this early growth stage [7], although armor is absent in the even smaller specimen of *Propanolosaurus* [59].

## Discussion

Ankylosauria is traditionally divided into two families, Ankylosauridae and Nodosauridae, which are distinct in many features [21]. A third group, Polacanthinae [60] or Polacanthidae [5], has been proposed, and it is normally defined as all ankylosaurians more closely related to *Gastonia* than to either *Edmontonia* or *Euoplocephalus* [5]. However, some phylogenetic analyses, including ours (see below) do not recover this group as a separate clade [1], [10], [26], and here we follow traditional ankylosaurian taxonomy in our discussion.

*Chuanqilong chaoyangensis* possesses many ankylosaurid features, including cheek teeth with a strongly swollen tooth crown with a weak cingulum, a long deltopectoral crest that extends for more than half of humeral length, a straight lateral margin of the preacetabular process, a very short postacetabular process that is shorter than the length of the acetabulum, a slender ischial shaft that is curved slightly ventrally, and a distally located fourth trochanter. However, it lacks several features shared by derived ankylosaurids, such as the presence of a tail club. This character combination suggests that *Chuanqilong chaoyangensis* represents a basal ankylosaurid.

**Figure 11:**
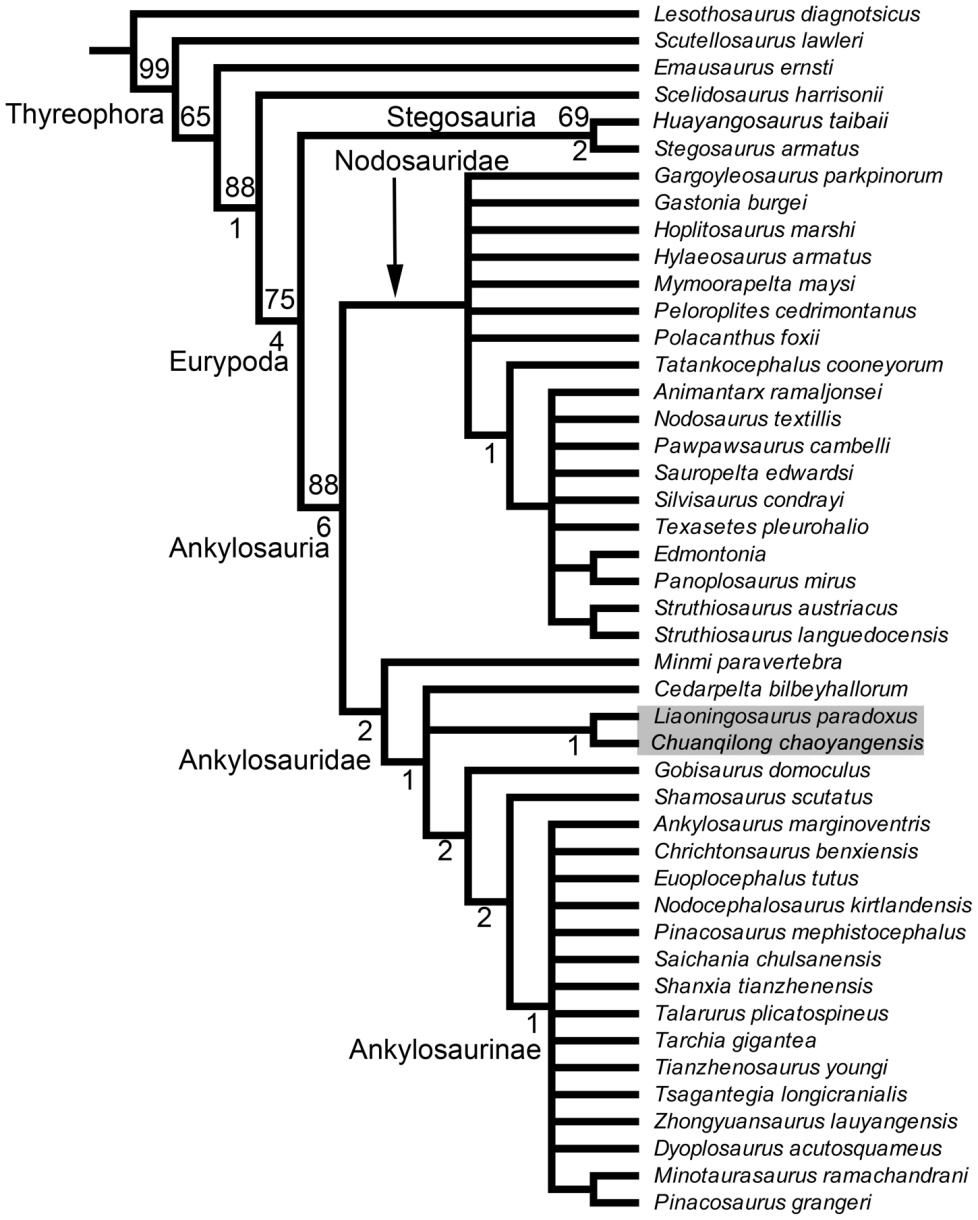
Derivative strict reduced consensus tree of ankylosaurian relationships. *Zhejiangosaurus*, *Niobrarasaurus*, *Hungarosaurus*, *Actarctopelta*, *Anoplosaurus*, *Polacathus rodgwickensis*, and *Stegopelta* were pruned *a posteriori* to improve resolution. Values above nodes are bootstraps, and values below nodes are Bremer support values. See text for further details. [planned for page width].

In order to confirm our hypothesis regarding the systematic position of *Chuanqilong chaoyangensis*, we conducted a phylogenetic analysis by adding *Chuanqilong chaoyangensis* to a recently published dataset on ankylosaurian phylogenetic relationships [10]. Our analysis produced 15902 most parsimonious trees (MPTs), with tree lengths of 542 steps (Consistency Index  = 0.34, Retention Index  = 0.66). The strict consensus tree (not shown) of these 15902 MPTs lacks resolution, with the only clear result being recovery of ankylosaurid monophyly. A reduced consensus tree was calculated a posteriori which excluded seven wildcard taxa (*Zhejiangosaurus*, *Niobrarasaurus*, *Hungarosaurus*, *Antarctopelta*, *Anoplosaurus*, *Polacanthus rudgwickensis*, and *Stegopelta*) [61], and this shows considerably greater resolution (Fig. 11).

Our results indicate that *Chuanqilong chaoyangensis* is a basal ankylosaurid and that it is the sister taxon of the sympatric *Liaoningosaurus*. However, only two unambiguous synapomorphies support this relationship: presence of an antorbital fossa or fenestra ([10]: character 1) and scapula glenoid oriented ventrally ([10]: character 121).

It should be noted that both *Chuanqilong chaoyangensis* and *Liaoningosaurus paradoxus* are represented by specimens at a relatively early ontogenetic stage, although the much larger size, relatively smaller orbit, and higher tooth count of *Chuanqilong* suggest that the latter is at a more advanced ontogenetic stage. Although *Chuanqilong* and *Liaoningosaurus* are sister taxa, they can be distinguished on the basis of the following characters, which are probably not ontogenetically variable:

1. Cheek tooth crown morphology. In *Chuanqilong*, the cheek teeth are relatively small compared to the skull, and there are more than 20 maxillary teeth, whereas in *Liaoningosaurus*, the cheek teeth are significantly larger in comparison to the skull, and there are only approximately 10 maxillary teeth. Although Xu et al. [7] noted that the low tooth number of *Liaoningosaurus* may be due to its juvenile status, the ontogenetic variation in teeth number among all known ankylosaurians is less than 10. For example, the variation in *Euoplocephalus* and *Pinacosaurus* cheek teeth number is five and three, respectively [28]. Additionally, the cheek tooth crowns of *Chuanqilong* bear small denticles and cusps, with approximately 12 denticles per tooth, whereas in *Liaoningosaurus*, the denticles are large cusps that are also relatively large with respect to the size of the tooth crown, and there are approximately seven denticles per tooth. The tooth crowns of *Liaoningosaurus* are similar to those of another ankylosaurid from Liaoning Province, *Crichtonsaurus bohlini*, but differ from those of *Chuanqilong*.
2. The proximal end of the humerus is strongly expanded in comparison to humeral length in *Chuanqilong* (the ratio of proximal width to whole length is 0.51), whereas it is only moderately expanded in *Liaoningosaurus* (the ratio of proximal width to whole length is 0.38). Additionally, the distal end of the deltopectoral crest extends for more than half of the length of the humerus in *Chuanqilong*, as in typical ankylosaurids, whereas in *Liaoningosaurus*, the deltopectoral crest is less developed and does not extend to the mid-length of the humerus, as in other nodosaurids.
3. The lateral edge of the ilium is straight or slightly convex in ventral view in *Chuanqilong*, whereas it is slightly concave above the acetabulum in *Liaoningosaurus*.
4. The ischial shaft of *Chuanqilong* has a constriction at mid-length and tapers distally, whereas the ischial shaft is relatively equal in width along the whole length and slightly expanded at the distal end in *Liaoningosaurus*.
5. The ratios of metatarsus to metacarpus length in *Chuanqilong* is substantially less than that of *Liaoningosaurus* (Fig. 5F, G). The metatarsus is more than twice the length of the metacarpus in *Liaoningosaurus* and this is probably an autapomorphy of this taxon [7]. It is unknown whether the ratio of metatarsus to metacarpus length changes during ontogeny. However, the metatarsus is less than twice the length of the metacarpus in the hatchling *Propanoplosaurus* ([59]: Fig. 5).
6. The pedal unguals of *Chuanqilong* are widest at a point approximately one-third of the distance from the proximal end and are slightly constricted at the proximal end, whereas the pedal unguals are sub-triangular and widest at the proximal end in *Liaoningosaurus* [7] and *Dyoplosaurus* [37].

Several juvenile ankylosaurians have been recognized and provide important ontogenetic information [7], [26], [42], [51], [52], [62]. These studies indicate that some features used for species diagnosis are probably under ontogenetic control, such as some fusion characters, including fusion of the scapula and coracoid, fusion of the calcaneum and astragalus, and fusion of the cranial and greater trochanters.

Ontogenetic variation may affect phylogenetic reconstruction (e.g. [63]). Many derived features found in adult specimens are rudimentary or undeveloped in juvenile specimens, making the latter appear more basal than adult individuals in phylogenetic analyses. Therefore, ideally, ontogenetically variable characters should be excluded from phylogenetic analysis or such analyses should be based upon adult specimens only. However, many ankylosaurians are only partially preserved and it has been difficult to document their ontogenetic variation. *Euoplocephalus* and *Pinacosaurus*, which are known from multiple individuals, may provide more insights into this problem, but the taxonomy of *Euoplocephalus* has been controversial and many formerly referred specimens are now thought to represent other distinct taxa [31]. In order to retain as many taxa in our analysis as possible, we were unable to exclude ontogenetic characters from our phylogenetic analysis. Further ontogenetic precision could be gained from aging individuals using bone histology, which has not been widely applied to ankylosaurians. As *Liaoningosaurus* and *Chuanqilong* are represented by juvenile specimens only, more material, especially adult specimens, will help to further elucidate their phylogenetic relationships.

*Chuanqilong* was moderate in size compared with other known ankylosaurians (Table 2). However, it still larger than adult Jurassic ankylosaurians, including *Mymoorapelta* and *Gargoyleosaurus* [18], [19]. The juvenile *Chuanqilong* is similar in size to most Cretaceous ankylosaurians, including adult *Hungarosaurus* [55] and *Europelta* [56], but is smaller than *Cedarpelta* (7.5–8.5 m: [20]) and *Polacanthus* (5–7 m: [53]). However, as the holotype of *Chuangqilong* is not fully-grown, based on the above-mentioned features, this suggests that the adults of this taxon may have been among the largest ankylosaurians. This suggests in turn that ankylosaurs has already evolved large size by the late Early Cretaceous. Bone histology should be used in future to gain a more accurate understanding of the ontogenetic age of this specimen.

## Supporting Information

Text S1 **Updated character scores for** ***Chuanqilong*** **, and additional scores for** ***Liaoningosaurus*** **.**
(DOC)

## References

[1] Vickaryous MK, Maryańska T, Weishampel DB (2004) Ankylosauria. In: Weishampel DB, Dodson P, Osmóska H, editors.The Dinosauria Second Edition. University of California Press. Berkeley. pp. 363–392.

[2] Dong Z-M (1993) An ankylosaur (ornithischian dinosaur) from the Middle Jurassic of the Junggar Basin, China. Vertebrata PalAsiatica 31: 257–266.

[3] Dong Z-M (2001) Primitive armored dinosaur from the Lufeng Basin, China. In: Tanke DH, Carpenter K, editors. Mesozoic vertebrate life: Indiana University Press. pp. 237–243.

[4] Galton PM (1983) Armored dinosaurs (Ornithischia: Ankylosauria) from the Middle and Upper Jurassic of Europe. Palaeontographica, Abteilung A 182: 1–25.

[5] Carpenter K (2001) Phylogenetic analysis of the Ankylosauria. In: Carpenter K, editor. The armored dinosaurs: Indiana University Press, Bloomington. pp. 455–483.

[6] Carpenter K, Miles C, Cloward K (1998) Skull of a Jurassic ankylosaur (Dinosauria). Nature 393: 782–783.

[7] Xu X, Wang X-L, You H-L (2001) A juvenile ankylosaur from China. Naturwissenschaften 88: 297–300. 11544897 10.1007/s001140100233

[8] Dong Z-M (2002) A new armored dinosaur (Ankylosauria) from Beipiao Basin, Liaoning Province, Northeastern China. Vertebrata PalAsiatica 40: 276–285.

[9] Lü J-C, Ji Q, Gao Y-B, Li Z-X (2007) A new species of the ankylosaurid dinosaur *Crichtonsaurus* (Ankylosauridae: Ankylosauria) from the Cretaceous of Liaoning Province, China. Acta Geologica Sinica (English Edition) 81: 883–897.

[10] Thompson RS, Parish JC, Maidment SC, Barrett PM (2012) Phylogeny of the ankylosaurian dinosaurs (Ornithischia: Thyreophora). Journal of Systematic Palaeontology 10: 301–312.

[11] Goloboff PA, Farris JS, Nixon KC (2008) TNT, a free program for phylogenetic analysis. Cladistics 24: 774–786.

[12] Owen R (1842) Report on British fossil reptiles, part II. Reports of the British Association for the Advancement of Science 11: 60–204.

[13] Seeley HG (1887) On the classification of the fossil animals commonly named Dinosauria. Proceedings of the Royal Society of London 43: 165–171.

[14] Nopcsa FB (1915) The dinosaurs of the Transylvanian Province in Hungary. Communications of the Yearbook of the Royal Hungarian Geological Institute 23: 1–26.

[15] Osborn HF (1923) Two Lower Cretaceous dinosaurs of Mongolia. American Museum Novitates 95: 1–10.

[16] Brown B (1908) The Ankylosauridae, a new family of armored dinosaurs from the Upper Cretaceous. Bulletin of the American Museum of Natural History 24: 187–201.

[17] He H-Y, Wang X-L, Zhou Z-H, Wang F, Boven A, et al (2004) Timing of the Jiufotang Formation (Jehol Group) in Liaoning, northeastern China, and its implications. Geophysical Research Letters 31: L12605.

[18] Kirkland JI, Carpenter K (1994) North America's first pre-Cretaceous ankylosaur (Dinosauria) from the Upper Jurassic Morrison Formation of western Colorado. Brigham Young University Geology Studies 40: 25–42.

[19] Kilbourne B, Carpenter K (2005) Redescription of *Gargoyleosaurus parkpinorum*, a polacanthid ankylosaur from the Upper Jurassic of Albany County, Wyoming. Neues Jahrbuch für Geologie und Paläontologie, Abhandlungen 237: 111–160.

[20] Carpenter K, Kirkland JI, Burge D, Bird J (2001) Disarticulated skull of a new primitive ankylosaurid from the Lower Cretaceous of eastern Utah. In: Carpenter K, editor. The armored dinosaurs: Indiana University Press, Bloomington, Indiana. pp. 211–238.

[21] Coombs WP Jr (1978) The families of the ornithischian dinosaur order Ankylosauria. Palaeontology 21: 143–170.

[22] Carpenter K, Hayashi S, Kobayashi Y, Maryańska T, Barsbold R, et al (2011) *Saichania chulsanensis* (Ornithischia, Ankylosauridae) from the Upper Cretaceous of Mongolia. Palaeontographica, Abteilung A 294: 1–61.

[23] Miles CA, Miles CJ (2009) Skull of *Minotaurasaurus ramachandrani*, a new Cretaceous ankylosaur from the Gobi Desert. Current Science 96: 65–70.

[24] Molnar RE (1996) Preliminary report a new ankylosaur from the Early Cretaceous of Queensland, Australia. Memoirs of the Queensland Museum 39: 653–668.

[25] Coombs WP Jr, Maryańska T (1990) Ankylosauria. In: Weishampel DB, Dodson P, Osmólska H, editors. The Dinosauria: University of California Press. Berkeley. pp. 456–483.

[26] Hill RV, Witmer LM, Norell MA (2003) A new specimen of *Pinacosaurus grangeri* (Dinosauria: Ornithischia) from the Late Cretaceous of Mongolia: ontogeny and phylogeny of ankylosaurs. American Museum Novitates 3395: 1–29.

[27] Vickaryous MK, Russell AP (2003) A redescription of the skull of *Euoplocephalus tutus* (Archosauria: Ornithischia): a foundation for comparative and systematic studies of ankylosaurian dinosaurs. Zoological Journal of the Linnean Society 137: 157–186.

[28] Carpenter K (2004) Redescription of *Ankylosaurus magniventris* Brown 1908 (Ankylosauridae) from the Upper Cretaceous of the Western Interior of North America. Canadian Journal of Earth Sciences 41: 961–986.

[29] Carpenter K, Breithaupt B (1986) Latest Cretaceous occurrence of nodosaurid ankylosaurs (Dinosauria, Ornithischia) in western North America and the gradual extinction of the dinosaurs. Journal of Vertebrate Paleontology 6: 251–257.

[30] Molnar RE (2001) Armor of the small ankylosaur *Minmi*. In: Carpenter K, editor. The armored dinosaurs: Indiana University Press, Bloomington. pp. 341–362.

[31] Arbour VM, Currie PJ (2013) *Euoplocephalus tutus* and the diversity of ankylosaurid dinosaurs in the Late Cretaceous of Alberta, Canada, and Montana, USA. PLoS ONE 8: e62421. 23690940 10.1371/journal.pone.0062421 PMC3648582

[32] Godefroit P, Pereda Suberbiola X, Li H, Dong Z-M (1999) A new species of the ankylosaurid dinosaur *Pinacosaurus* from the Late Cretaceous of Inner Mongolia (PR China). Bulletin-Institut Royal des Sciences Naturelles de Belgique Sciences de la Terre 69: 17–36.

[33] Maleev EA (1956) Armored dinosaurs from the Upper Cretaceous of Mongolia. Trudy Paleontologičeskogo Instituta Akademii Nauk SSSR 62: 51–91 [in Russian].

[34] Tumanova T (1987) The armored dinosaurs of Mongolia. Transactions of the joint Soviet-Mongolian Palaeontological Expedition 32: 1–76 [in Russian].

[35] Arbour VM, Lech-Hernes NL, Guldberg TE, Hurum JH, Currie PJ (2013) An ankylosaurid dinosaur from Mongolia with in situ armour and keratinous scale impressions. Acta Palaeontologica Polonica 58: 55–64.

[36] Pang Q-Q, Cheng Z-W (1998) A new ankylosaur of Late Cretaceous from Tianzhen, Shanxi. Progress in Natural Science 8: 326–334 [in Chinese]

[37] Arbour VM, Burns ME, Sissons RL (2009) A redescription of the ankylosaurid dinosaur *Dyoplosaurus acutosquameus* Parks, 1924 (Ornithischia: Ankylosauria) and a revision of the genus. Journal of Vertebrate Paleontology 29: 1117–1135.

[38] Burns ME, Sullivan RM (2011) The tail club of *Nodocephalosaurus kirtlandensis* (Dinosauria: Ankylosauridae), with a review of ankylosaurid tail club morphology and biostratigraphy. New Mexico Museum of Natural History Bulletin 53: 179–186.

[39] Vickaryous MK, Russell AP, Currie PJ, Zhao X-J (2001) A new ankylosaurid (Dinosauria: Ankylosauria) from the Lower Cretaceous of China, with comments on ankylosaurian relationships. Canadian Journal of Earth Sciences 38: 1767–1780.

[40] Xu L, Lu J, Zhang X, Jia S, Hu W, et al (2007) New nodosaurid ankylosaur from the Cretaceous of Ruyang, Henan Province. Acta Geologica Sinica 81: 433–438.

[41] Molnar RE (1980) An ankylosaur (Ornithischia: Reptilia) from the Lower Cretaceous of southern Queensland. Memoirs of the Queensland Museum 20: 77–87.

[42] Pereda Suberbiola X, Barrett PM (1999) A systematic review of ankylosaurian dinosaur remains from the Albian-Cenomanian of England. Special Papers in Palaeontology 60: 177–208.

[43] Burns ME, Sullivan RM (2011) A new ankylosaurid from the Upper Cretaceous Kirtland Formation, San Juan Basin, with comments on the diversity of ankylosaurids in New Mexico. New Mexico Museum of Natural History and Science Bulletin 53: 169–178.

[44] Carpenter K, Bartlett J, Bird J, Barrick R (2008) Ankylosaurs from the Price River Quarries, Cedar Mountain Formation (Lower Cretaceous), east-central Utah. Journal of Vertebrate Paleontology 28: 1089–1101.

[45] Maryańska T (1977) Ankylosauridae (Dinosauria) from Mongolia. Palaeontologia Polonica 37: 85–151.

[46] Currie PJ, Badamgarav D, Koppelhus EB, Sissons R, Vickaryous MK (2011) Hands, feet, and behaviour in *Pinacosaurus* (Dinosauria: Ankylosauridae). Acta Palaeontologica Polonica 56: 489–504.

[47] Carpenter K, DiCroce T, Kinneer B, Simon R (2013) Pelvis of *Gargoyleosaurus* (Dinosauria: Ankylosauria) and the origin and evolution of the ankylosaur pelvis. PloS one 8: e79887. 24244573 10.1371/journal.pone.0079887 PMC3828194

[48] Garcia G, Pereda Suberbiola X (2003) A new species of *Struthiosaurus* (Dinosauria: Ankylosauria) from the Upper Cretaceous of Villeveyrac (southern France). Journal of Vertebrate Paleontology 23: 156–165.

[49] Lü J-C, Jin X-S, Sheng Y-M, Li Y-H, Wang G-P, et al (2007) New nodosaurid dinosaur from the Late Cretaceous of Lishui, Zhejiang Province, China. Acta Geologica Sinica (English Edition) 81: 344–350.

[50] Coombs WP Jr (1979) Osteology and myology of the hindlimb in the Ankylosauria (Reptillia, Ornithischia). Journal of Paleontology: 666–684.

[51] Coombs WP Jr (1986) A juvenile ankylosaur referable to the genus *Euoplocephalus* (Reptilia, Ornithischia). Journal of Vertebrate Paleontology 6: 162–173.

[52] Jacobs LL, Winkler DA, Murry PA, Maurice JM, Carpenter K, et al.. (1996) A nodosaurid scuteling from the Texas shore of the Western Interior Seaway. In: Carpenter K, Hirsch K, Horner J, editors.Dinosaur eggs and babies.New York: Cambridge University Press. pp. 337–346.

[53] Pereda-Suberbiola J (1994) *Polacanthus* (Ornithischia, Ankylosauria), a trans-Atlantic armoured dinosaur from the Early Cretaceous of Europe and North America. Palaeontographica, Abteilung A 232: 133–159.

[54] Coombs WP Jr (1995) A nodosaurid ankylosaur (Dinosauria: Ornithischia) from the Lower Cretaceous of Texas. Journal of Vertebrate Paleontology 15: 298–312.

[55] Ösi A, Makádi L (2009) New remains of *Hungarosaurus tormai* (Ankylosauria, Dinosauria) from the Upper Cretaceous of Hungary: skeletal reconstruction and body mass estimation. Paläontologische Zeitschrift 83: 227–245.

[56] Kirkland JI, Alcalá L, Loewen MA, Espílez E, Mampel L, et al (2013) The basal nodosaurid ankylosaur *Europelta carbonensis* n. gen., n. sp. from the Lower Cretaceous (Lower Albian) Escucha Formation of Northeastern Spain. PLoS ONE 8: e80405. 24312471 10.1371/journal.pone.0080405 PMC3847141

[57] Kirkland JI, Carpenter K, Hunt A, Scheetz RD (1998) Ankylosaur (Dinosauria) specimens from the Upper Jurassic Morrison Formation. Modern Geology 23: 145–177.

[58] Penkalski P (2001) Variation in specimens referred to *Euoplocephalus tutus*. In: Carpenter K, editor.The Armored Dinosaurs.Bloomington: Indiana University Press. pp. 299–317.

[59] Stanford R, Weishampel DB, Deleon VB (2011) The First Hatchling Dinosaur Reported from the Eastern United States: *Propanoplosaurus marylandicus* (Dinosauria: Ankylosauria) from the Early Cretaceous of Maryland, USA. Journal of Paleontology 85: 916–924.

[60] Kirkland J (1998) A polacanthine ankylosaur (Ornithischia: Dinosauria) from the Early Cretaceous (Barremian) of eastern Utah. Lower and Middle Cretaceous Terrestrial Ecosystems New Mexico Museum of Natural History and Science Bulletin 14: 271–281.

[61] Wilkinson M (1994) Common cladistic information and its consensus representation: reduced Adams and reduced cladistic consensus trees and profiles. Systematic Biology 43: 343–368.

[62] Maryańska T (1971) New data on the skull of *Pinacosaurus grangeri* (Ankylosauria). Palaeontologia Polonica 25: 45–53.

[63] Campione NE, Brink KS, Freedman EA, McGarrity CT, Evans DC (2013) ‘Glishades ericksoni’, an indeterminate juvenile hadrosaurid from the Two Medicine Formation of Montana: implications for hadrosauroid diversity in the latest Cretaceous (Campanian-Maastrichtian) of western North America. Palaeobiodiversity and Palaeoenvironments 93: 65–75.

[64] Xu X, Norell MA (2006) Non-avian dinosaur fossils from the Lower Cretaceous Jehol Group of western Liaoning, China. Geological Journal 41: 419–437.

[65] Carpenter K, Dilkes D, Weishampel DB (1995) The dinosaurs of the Niobrara Chalk Formation (Upper Cretaceous, Kansas). Journal of Vertebrate Paleontology 15: 275–297.

[66] Arbour VM, Currie PJ (2013) The taxonomic identity of a nearly complete ankylosaurid dinosaur skeleton from the Gobi Desert of Mongolia. Cretaceous Research 46: 24–30.

[67] Carpenter K (1984) Skeletal reconstruction and life restoration of *Sauropelta* (Ankylosauria: Nodosauridae) from the Cretaceous of North America. Canadian Journal of Earth Sciences 21: 1491–1498.

