# Supplementary material for: A New Basal Ankylosaurid (Dinosauria: Ornithischia) from the Lower Cretaceous Jiufotang Formation of Liaoning Province, China
Source: PLoS One. 2014 Aug 13;9(8):e104551. doi: 10.1371/journal.pone.0104551 (PMC4131922; doi:10.1371/journal.pone.0104551)
Supplement: Text S1 — Updated character scores for Chuanqilong , and additional scores for Liaoningosaurus . (DOC) [file pone.0104551.s001.doc]

**Text S1**. Updated character scores for *Chuanqilong*, and additional scores for *Liaoningosaurus***.** The full data matrix used for the analysis conducted herein is available as Online Supplementary Information and the character list is published in Thompson et al. (2012).

1 11 21 31 41

*Chuanqilong* 1??1?????? ?????????? ???????11? ??101?111? ??????????

51 61 71 81 91

*Chuanqilong* ?????????? ???11???11 101?0????? ?????????? ????1???10

101 111 121 131 141

*Chuanqilong* ?????????? 010?1????0 100001?001 00??011100 11????0021

151 161

*Chuanqilong* 1?11102??? ??????00?0

New scores for *Liaoningosaurus*: character 1 (1), 66 (0), 68 (?), 71 (0), 72(0), 99 (1), 105 (0), 117(?), 120(0), 131(0), 133(?), 134(1), 144(?), 148(0), 154(1), 155(1).
